# Brain Anatomical Mediators of *GRIN2B* Gene Association with Attention/Hyperactivity Problems: An Integrated Genetic-Neuroimaging Study

**DOI:** 10.3390/genes12081193

**Published:** 2021-07-30

**Authors:** Maria Nobile, Eleonora Maggioni, Maddalena Mauri, Marco Garzitto, Sara Piccin, Carolina Bonivento, Roberto Giorda, Rossano Girometti, Barbara Tomasino, Massimo Molteni, Franco Fabbro, Paolo Brambilla

**Affiliations:** 1 Child Psychopathology Unit, Scientific Institute, IRCCS Eugenio Medea, 22040 Bosisio Parini, Italy; maria.nobile@lanostrafamiglia.it (M.N.); Maddalena.mauri@lanostrafamiglia.it (M.M.); massimo.molteni@lanostrafamiglia.it (M.M.); 2 Department of Neurosciences and Mental Health, Fondazione IRCCS Ca’ Granda Ospedale Maggiore Policlinico, 20122 Milan, Italy; eleonora.maggioni@policlinico.mi.it; 3 School of Medicine and Surgery, University of Milano-Bicocca, 20126 Milan, Italy; 4 Scientific Institute, IRCCS E. Medea, San Vito al Tagliamento, 33170 Pordenone, Italy; marco.garzitto@uniud.it (M.G.); carolina.bonivento@lanostrafamiglia.it (C.B.); barbara.tomasino@lanostrafamiglia.it (B.T.); franco.fabbro@uniud.it (F.F.); 5 Unit of Psychiatry, Department of Medicine (DAME), University of Udine, 33100 Udine, Italy; sara.piccin@gmail.com; 6 Molecular Biology Laboratory, Scientific Institute, IRCCS Eugenio Medea, 22040 Bosisio Parini, Italy; roberto.giorda@lanostrafamiglia.it; 7 Institute of Radiology, Department of Medicine, University of Udine, 33100 Udine, Italy; rossano.girometti@uniud.it; 8 Department of Pathophysiology and Transplantation, University of Milan, 20122 Milan, Italy; 9 Department of Psychiatry and Behavioral Sciences, McGovern Medical School, The University of Texas Health Science Center at Houston, Houston, TX 77030, USA

**Keywords:** imaging genetics, magnetic resonance imaging, neurodevelopment, mediation, gray matter volume, cortical surface area, cortical thickness

## Abstract

This study aims to investigate the genetic and neural determinants of attention and hyperactivity problems. Using a proof-of-concept imaging genetics mediation design, we explore the relationship between the glutamatergic *GRIN2B* gene variants and inattention/hyperactivity with neuroanatomical measures as intermediates. Fifty-eight children and adolescents were evaluated for behavioral problems at three time points over approximately 7 years. The final assessment included blood drawing for genetic analyses and 3T magnetic resonance imaging. Attention/hyperactivity problems based on the Child Behavior Checklist/6-18, six *GRIN2B* polymorphisms and regional cortical thickness, and surface area and volume were estimated. Using general linear model (GLM) and mediation analyses, we tested whether *GRIN2B* exerted an influence on stable inattention/hyperactivity over development, and to what extent this effect was mediated by brain morphology. GLM results enlightened the relation between *GRIN2B* rs5796555-/A, volume in the left cingulate isthmus and inferior parietal cortices and inattention/hyperactivity. The mediation results showed that rs5796555-/A effect on inattention/hyperactivity was partially mediated by volume in the left isthmus of the cingulate cortex, suggesting a key role of this region in translating glutamatergic *GRIN2B* variations to attention/hyperactivity problems. This evidence can have important implications in the management of neurodevelopmental and psychiatric disorders.

## 1. Introduction

Attention and hyperactivity problems—which are core symptoms of attention deficit/hyperactivity disorder (ADHD) but are also expressed in other developmental internalizing and externalizing disorders (e.g., anxiety, depression, oppositional defiant, disruptive mood dysregulation disorders)—are complex behavior traits with a multifactorial etiology: genetic, epigenetic, and environmental factors influence their development and persistence [1,2]. 

Literature evidence suggests high stability for both attention problems and hyperactivity-impulsivity traits, but their phenotypes seem to follow different developmental trajectories. Indeed, gender, pharmacologic, or psychosocial treatment and environment might influence their manifestation over time [3]. Twin studies on developmental aged cohorts found a high heritability of attention problems, between 70 and 75%. This genetic influence is stable from 3 to 12 years of age, with trait correlations estimated between 0.40 and 0.70 [4,5]. Regarding ADHD diagnosis, heritability is estimated at 70% [6,7].

One of the most frequently investigated genes in populations with attention and hyperactivity problems is the glutamatergic *GRIN2B*, a moderately large gene located on chromosome 12p13.1, comprising 13 exons and spanning a genomic region of approximately 400 kb [8].

*GRIN2B* codes for the Glun2B subunit of N-Methyl-D-Aspartate (NMDA) receptor, which mediates the slow Ca^+^ component of excitatory synaptic transmission in the central nervous system. Glun2B is highly expressed prenatally [9] and plays a central role in brain development, synaptic plasticity, and long-term potentiation [10,11].

These molecular mechanisms are crucial in the development of different cognitive functions, such as memory, learning, and attention [12,13]. In fact, *GRIN2B* variants have been associated not only with ADHD [8], but also with cognitive deficits in heterogeneous neurodevelopmental and psychiatric disorders, including autism spectrum disorders, Alzheimer’s and Parkinson’s diseases, bipolar disorder, and schizophrenia [14,15,16,17,18]. 

In recent years, the joint analysis of genomic and neuroimaging data, known as imaging genetics, has provided the opportunity to get a more complete knowledge of how genetic and neurobiological factors interplay to determine behaviors [19]. Imaging genetics research sets its roots on the evidence of a close association of genetics with brain structure and function, in accord with the notion that brain physiology is etiologically closer to molecular biology than behavior [20]. In this line, studies of genetic effects on behavior should account for neuroimaging parameters as intermediate phenotypes, which may influence the link between genetic variants and complex behaviors.

In Imaging genetics literature, there is evidence of an association between variants in glutamate system genes, including *GRIN2B* polymorphisms, and neuroimaging phenotypes in healthy and clinical populations [21,22]. Recent magnetic resonance studies have suggested a link between *GRIN2B* variants and abnormal glutamatergic neurotransmission and brain volume in children and adolescents with obsessive compulsive [23] and alcohol use [24] disorders. Although ADHD has been shown to share symptoms and risk factors with these disorders [25,26], the complex relationships between the *GRIN2B* gene, brain, and inattention/hyperactivity traits remain largely unknown.

In this pilot work, for the first time, we explore the possible link between *GRIN2B* marker variants, changes in brain morphology, and attention/hyperactivity problems in a juvenile sample. Through an exploratory mediation design, we intend to verify whether *GRIN2B* polymorphisms influence attention and hyperactivity phenotypes and, if yes, if such an effect is mediated (and in what proportions) by brain morphology.

## 2. Materials and Methods

### 2.1. Longitudinal Study Protocol

The present study is part of the Genesis Project, an imaging genetics longitudinal study conducted to identify and understand developmental trajectories of mental disorders in children and adolescents.

The Genesis Project involved a clinical sample of children and adolescents who were referred for emotional and behavioral problems at the Scientific Institute IRCCS Eugenio Medea (Italy) [27,28]. The research design involved three observations of the recruited cohort. At Wave 0, a demographic and clinical assessment of the subjects was performed, including the administration of the Kiddie Schedule for Affective Disorders and Schizophrenia-Present and Lifetime Version [29], the Child Behavior Checklist (CBCL/6-18) [30], the Barratt’s Simplified Measure of Social Status [31] and the Wechsler Intelligence Scale for Children [32]. At Waves 1 and 2, subjects were re-evaluated for emotional and behavioral problems using the CBCL/6-18. At Wave 2, the assessment included blood drawing for genetic analyses and a magnetic resonance imaging (MRI) session. More details are reported in the following sections.

### 2.2. Subjects

The participants’ sociodemographic, clinical and cognitive characteristics are reported in Table 1. The sample included 58 unrelated patients (42 males and 16 females, aged 8.78 ± 2.43 years) who took part in the three waves of the Genesis Project. In the recruited sample, Waves 1 and 2 were conducted 5.74 ± 1.66 and 7.39 ± 1.65 years after Wave 0, respectively.

Exclusion criteria were diagnoses of Autism Spectrum Disorder or Intellectual Disability, neurological diseases (including epilepsy and traumatic brain injuries), severe sensory and linguistic comprehension deficits.

The study protocol was approved by the Research Ethical Committee of our Scientific Institute and performed in accordance with the Declaration of Helsinki. Parents’ written informed consent to the study was obtained for all participants.

### 2.3. Behavioral and Clinical Measures

#### Child Behavior Checklist (CBCL/6-18)

It is an empirically-based checklist of social competence and behavioral problems filled out by parents of children and adolescents aged 6–18 years. According to the Achenbach System of Empirically Based Assessment (ASEBA) [30], CBCL/6-18 items can be scored to obtain the following eight subscales: anxious/depressed, withdrawn/depressed, somatic complaints, rule-breaking, aggressive behavior, social, thought and attention problems. The parent responds along a 3-point scale where 0, 1, and 2 indicate that the behavior is not true, sometimes true or often true for the child, respectively. The psychometric stability of the CBCL/6-18 has been well established [33,34]. 

In this study, we employed the attention problems (AP) scale T scores (mean = 50; standard deviation = 10) based on the set of multicultural norms “group 2”, which applies to the normative sample of the Italian population as suggested by the multicultural supplement to the ASEBA manual. The AP scale consists of 11 items (e.g., cannot concentrate, cannot pay attention for long, is impulsive or acts without thinking) assessing both inattentive and hyperactive-impulsive symptoms. For the AP scale, the ASEBA identifies as normal scores below 65, as borderline scores between 65 and 70, and as clinical scores above 70. In the following analyses, we considered the mean score obtained in the AP scale across the three time points, which represents a stable measurement of this behavioral dimension over development.2.3.2. Kiddie Schedule for Affective Disorders and Schizophrenia for School-Age Children, Present and Lifetime Version (K-SADS-PL).

K-SADS-PL is a semi-structured diagnostic interview created to assess current and past episodes of psychopathology in children and adolescents according to DSM-III-R and DSM-IV criteria. 

### 2.4. Genotyping

Participants’ DNA was obtained from saliva samples collected and extracted using Oragene OG-500 kits (DNA Genotek, Ottawa, Canada). Amplification and sequencing of *GRIN2B* regions allowed us to type rs2268119 A/T, rs22161128 A/G, rs5796555 -/A, rs1012586 G/C, rs11609779 C/T, rs2192973 G/A Single Nucleotide Polymorphisms (SNPs).

Amplifications were performed in 10-μL reactions using JumpStart REDAccuTaq LA DNA polymerase (Sigma-Aldrich, St. Louis, MO, USA) and the following protocol: 30 s at 96 °C, 35 cycles of 15 s at 94 °C/20 s at 58 °C/30 s at 68 °C, 5 min final elongation time. Sequencing reactions were performed with a Big Dye Terminator Cycle Sequencing kit (Applied Biosystems, Monza, Italy) and run on ABI Prism 3130xl (Applied Biosystems, Monza, Italy) and 3500AV Genetic Analyzers (Applied Biosystems, Monza, Italy). 

**Figure 1:**
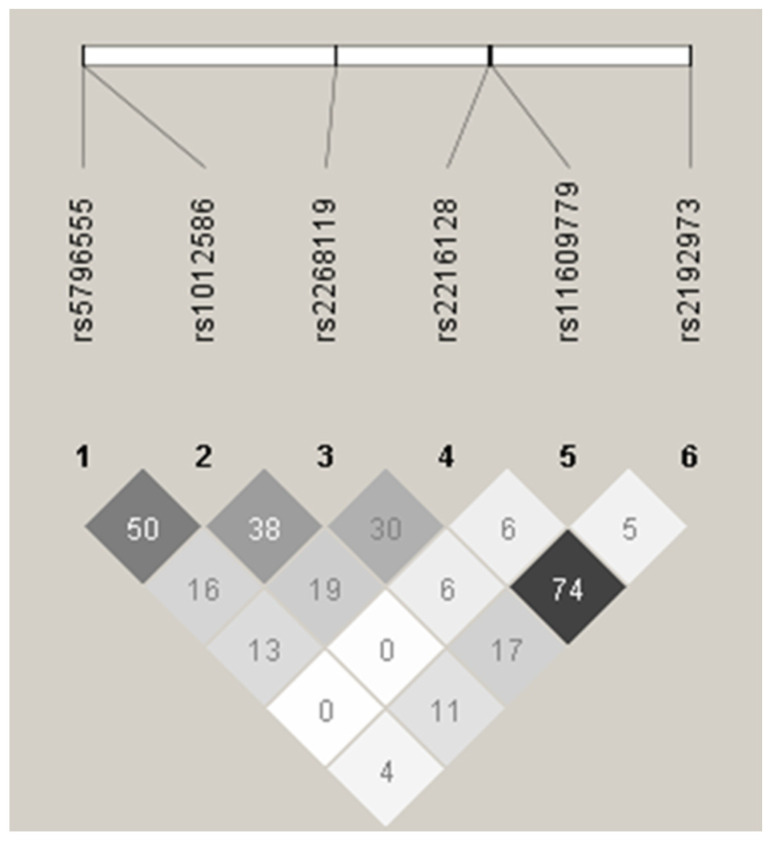
*GRIN2B* linkage disequilibrium. Haploview plot showing pairwise linkage disequilibrium (r^2^ values) for 6 SNPs of *GRIN2B* based on the sample’s genotypes.

Table 2 shows allelic frequencies and Hardy-Weinberg equilibrium (HWE) for the considered markers. Genotype distributions did not significantly deviate from HWE. No SNPs were therefore excluded from further analyses. The *GRIN2B* linkage disequilibrium structure (Figure 1) shows a squared correlation coefficient between 0.00 and 0.74. Genotypes were grouped into a two-level variable, each level representing the presence or absence of minor frequency alleles.

### 2.5. MRI Data Acquisition

Structural MRI data were acquired in the University Hospital of Udine (Udine, Italy) using a Philips Achieva 3.0 Tesla scanner (Philips Healthcare, Best, The Netherlands) equipped with an 8-channel head coil for radiofrequency transmission and reception. All images were obtained with a T1-weighted MPRAGE 3D TFE sequence, with the following parameters: echo time = 3.7 ms, repetition time = 8.1 ms, in-plane field of view = 240 × 240 mm^2^, in-plane matrix size = 240 × 240, 190 axial slices with no gap, voxel size = 1 mm^3^. 

### 2.6. MRI Data Processing

The MRI images were processed using the open-source Freesurfer software, v5.3.0 (http://surfer.nmr.mgh.harvard.edu/, downloaded on 8 March 2017) [35], which provides an accurate 3D reconstruction of the cerebral cortex. For each subject, starting from the T1-weighted image, Freesurfer performs a brain tissue segmentation and estimates the gray matter/white matter interface, which is used to model the cortical surfaces. In our study, the segmentation output and the reconstructed surfaces were visually inspected and corrected, if necessary, by a trained user.

The subject’s cortical model was parceled into regions of interest (ROIs) based on the Desikan-Killiany atlas [36] and cortical thickness (CT), cortical surface area (CSA), and gray matter volume (GMV) were estimated at the ROI level and used for the following analyses. 

### 2.7. Statistical Analyses

#### 2.7.1. General Linear Model (GLM) Analyses

In a set of preliminary GLM analyses based on in-house Matlab scripts (R2018b, The Mathworks, Inc. Natick, MA, USA), we investigated the relation among *GRIN2B* markers, brain morphology and inattention/hyperactivity. First, we evaluated the impact of *GRIN2B* SNPs on neuroanatomical parameters (design #1), *GRIN2B* SNPs on inattention/hyperactivity (mean CBCL/6-18 AP score over time) (design #2). We then selected the morphological parameters significantly influenced by the *GRIN2B* SNP/SNPs exerting a significant effect on attention/hyperactivity (intersection of results #1 and #2) and investigated their possible influence on the CBCL/6-18 AP variable (design #3). 

*GRIN2B* SNPs and regional morphological parameters were investigated one by one in separate models, i.e., we performed 1116 GLM analyses for design #1 (combination of 62 ROIs, three surface-based measures for each ROI and 6 *GRIN2B* SNPs) and 6 GLM analyses for design #2. In all GLM designs, age and gender were added as covariates to remove their possible contribution to the results. In designs #1 and #3, the total intracranial volume was used as a normalization factor when focusing on GMV, while the total surface area was used when focusing on CSA.

We made inference using double-sided t-tests, where the significance threshold was set to *p* = 0.05. In the GLM design #1 analyses, in order to limit false positive rates, a correction for multiple comparisons (MC) was applied (N = 37, 31 regions in each hemisphere + 6 *GRIN2B* markers).

The GLM results were examined to detect any joint relationships among *GRIN2B* SNPs, ROI parameters, and CBCL/6-18 AP score. The mutually related variables were used in the following mediation analysis, with the objective to check whether the causal effect of *GRIN2B* marker variants on attention/hyperactivity phenotype was mediated (and, if yes, in what proportions) by brain morphology.

#### 2.7.2. Mediation Analyses

**Figure 2:**
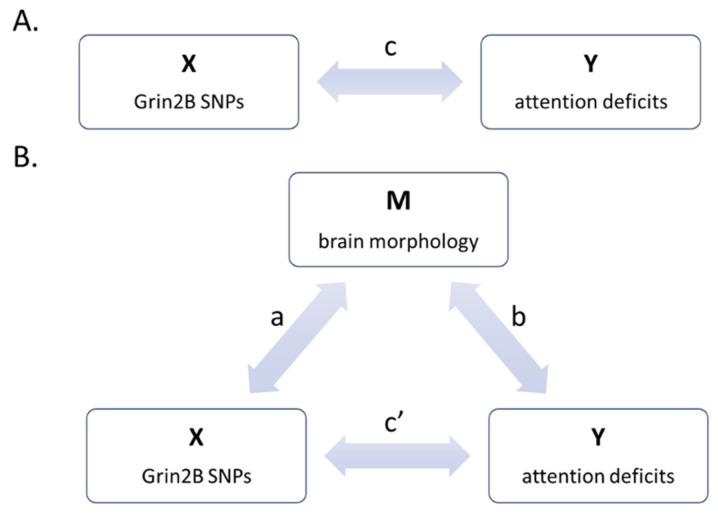
Mediation model design. (**A**) Model of direct effect of X on Y. (**B**) Mediation model, where X has both direct and indirect (through M) effects on Y.

Mediation was assessed using the open-source Bootstrap Regression Analysis of Voxelwise Observations (BRAVO) toolbox (https://sites.google.com/site/bravotoolbox/, downloaded on 5 March 2018) in Matlab. On each triad of selected variables, we designed a simple mediation model, where the *GRIN2B* SNP was the causal variable X, the CBCL/6-18 AP score the outcome Y, and the ROI morphological parameter the mediator M (Figure 2).

Provided that X significantly accounts for variability in both Y (path c) and M (path a), and M accounts for variability in Y when covarying for X (path b), M is the mediator of the X-Y relationship if the effect of X on Y substantially decreases when M is entered simultaneously with X as a predictor of Y (path c’). Further details on mediation models can be found in [37]. 

In our study, we used a mediation regression model with age and gender as covariates. The mediation significance was assessed through a permutation procedure with 5000 iterations. Before running the analyses, the values of X, Y, and M were normalized using the Z-score standardization. The strength of model path, for both observed data and bootstrap distributions, was assessed through Ordinary Least Square regression. Confidence intervals and *p*-values were then estimated using the bias corrected and accelerated formula described in [38]. For all model coefficients (a, b, ab, c, c’), the significance threshold was set to *p* = 0.05. Multiple comparison corrections were performed if appropriate, based on the number of mediation models applied.

## 3. Results

### 3.1. GLM Analyses

#### 3.1.1. Design #1. *GRIN2B* Effects on Neuroanatomy

The GLM statistics concerning *GRIN2B* effects on regional brain morphology are reported in Table 3. We detected significant associations between rs5796555-/A marker and regional GMV, and rs2268119A/T and rs2216128T/C markers and regional CSA (*p* < 0.05, MC corrected). On the contrary, no significant effects of *GRIN2B* markers on CT emerged.

Specifically, the less frequent allele “A” of marker rs5796555-/A was associated with GMV deficits in left isthmus of cingulate cortex, left precuneus, right caudal and rostral anterior cingulate cortex, right transverse temporal gyrus and bilateral rostral middle frontal gyrus, inferior parietal gyrus, middle temporal cortex and pars orbitalis. Notably, this effect was highly significant (*p* < 0.01, MC corrected) in regions of the right hemisphere, that is, the caudal and rostral anterior cingulate cortex and inferior parietal gyrus. As inferred from Table 3, the peak T statistics was observed in the caudal portion of the right anterior cingulate cortex.

We also found a negative association between the genotype carrying the less frequent allele “T” of marker rs2268119A/T and CSA in left lateral orbitofrontal cortex (*p* < 0.05, MC corrected) and right lateral occipital cortex (*p* < 0.01, MC corrected), and the genotype carrying the minor allele “C” of marker rs2216128G/C and CSA in the right isthmus of the cingulum (*p* < 0.05, MC corrected). None of the above brain features were affected by gender or age (*p* > 0.05).

#### 3.1.2. Design #2. *GRIN2B* Association with Attention/Hyperactivity Problems

The GLM analyses assessing the impact of *GRIN2B* markers on inattention/hyperactivity revealed a significant positive association between the genotype carrying the minor allele ‘A’ of marker rs5796555-/A and the mean CBCL/6-18 AP score (T(54) = 2.41, *p* < 0.05). No influences of gender or age on this score emerged (*p* > 0.05).

#### 3.1.3. Design #3. Neuroanatomy Effects on Attention/Hyperactivity Problems

In view of the results of designs #1 and #2, the neuroanatomy-attention GLM analyses were performed on the only brain morphological parameters influenced by *GRIN2B* marker rs5796555-/A (Table 3). We found that the CBCL/6-18 AP score was inversely proportional to GMV in the left isthmus of the cingulate cortex (T(54) = 2.67, *p* < 0.05) and in the right inferior parietal cortex (T(54) = 2.26, *p* < 0.05), suggesting a possible role of these regions as mediators of the effect of *GRIN2B* marker rs5796555-/A on inattention/hyperactivity.

### 3.2. Mediation Analyses

In view of the GLM results, two separate mediation analyses (MA1 and MA2) were performed to investigate the relationship among *GRIN2B* rs5796555-/A genotype (causal variable X), CBCL/6-18 AP score (outcome Y), and (i) GMV in the left isthmus of the cingulate cortex (mediator variable M1), (ii) GMV in the right inferior parietal cortex (mediator variable M2). No mediation analyses were performed on *GRIN2B* genotypes or brain features other than those specified due to the absence of the mediation model prerequisites.

The mediation model parameters, 95% confidence intervals (CI), and p-values are reported in Table 4. In line with preliminary GLM results, both mediation analyses confirmed a significant total effect of rs5796555-/A genotype on CBCL/6-18 AP score (c = 0.31, *p* < 0.05).

**Figure 3:**
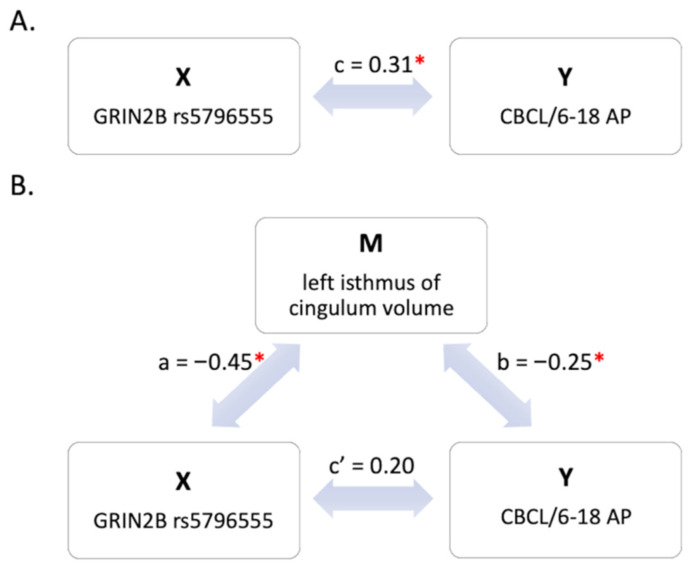
Mediation analysis 1 (MA1) results. (**A**) Model of direct effect of X on Y. (**B**) Mediation model, where X has both direct and indirect (through M) effects on Y. Significant pathways are highlighted with *. CBCL/6-18 AP: Child Behavior Checklist Attention Problems score.

As shown in Figure 3, MA1 results confirmed that genotypes carrying the minor allele A of rs5796555-/A were associated with GMV deficits in the left isthmus of the cingulate cortex (a = −0.45, *p* < 0.001), and in turn that such deficits (while regressing out rs5796555-/A effect) were linked to CBCL/6-18 AP score (b = −0.25, *p* < 0.05). After inclusion of M1 in the model, the direct effect of rs5796555-/A on CBCL/6-18 AP score was not significant (c’ = 0.20, *p* = 0.09), whereas its indirect effect through GMV in the left isthmus of cingulate cortex remained significant (ab = 0.11, *p* < 0.001). Specifically, 35.89% of the total rs5796555-/A effect on attention was mediated by GMV in this region.

On the contrary, MA2 showed that rs5796555-/A genotypes were linked to GMV in the right inferior parietal gyrus (a = −0.48, *p* < 0.001), but did not confirm a significant influence of the right inferior parietal deficits on attention/hyperactivity problems while controlling for rs5796555-/A contribution (b = −0.18, *p* = 0.08). The absence of a net effect of M2 on Y ruled out the investigation of any mediated effects of this variable. Of note, since only MA1 analysis was successfully conducted, no multiple comparison corrections were performed on MA1 analysis coefficients.

## 4. Discussion

In this preliminary work, an innovative genetic-neuroimaging-behavioral approach was adopted to investigate the potential causal effects of *GRIN2B* markers on developmental attention/hyperactivity problems through neuroanatomy. To this end, we genotyped 6 *GRIN2B* markers and measured brain morphological parameters and inattention/hyperactivity in a large clinical sample followed from childhood to adolescence. 

Our results confirm the presence of a causal chain of relationships among the three variables, showing that *GRIN2B* rs5796555-/A effects on inattention/hyperactivity over time is significantly mediated by volume in the left isthmus of the cingulate cortex. This unprecedented finding supports the hypothesis that *GRIN2B* variants affect the structure of key brain regions for executive functioning, ultimately exerting both direct and indirect impact on these developmental problems. This evidence offers new opportunities and translational pathways in the identification and management of attention/hyperactivity deficits in the delicate phase of neurodevelopment. 

### 4.1. *GRIN2B* Effect on Attention Deficits

To our knowledge, this is the first study on *GRIN2B* that considered multiple time measures of behavioral attention/hyperactivity problems. The use of the mean CBCL/6-18 AP score across three time points allowed us to smooth the variability due to different manifestations of this complex behavior from childhood to adolescence. In fact, the CBCL/6-18 AP scale assesses both inattentive and hyperactive-impulsive symptoms linked to ADHD, and longitudinal studies suggest that these dimensions may follow separate developmental trajectories and have different manifestations at different ages [3]. 

In line with previous studies, we found a significant relation between persistent attention deficits and the *GRIN2B* gene. Specifically, subjects carrying the minor allele ‘A’ of *GRIN2B* rs5796555-/A were characterized by a higher CBCL/6-18 AP score.

Our findings further confirm the crucial role of *GRIN2B* in behavioral functions. Given the importance of the Glun2B subunit of NMDA receptor for maturation and plasticity of the central nervous system, it is not surprising that over 60 variants of *GRIN2B* have been associated with heterogeneous neurodevelopmental and psychiatric disorders [16].

Previous literature reported evidence of the association of *GRIN2B* gene variants with attention deficits in the general population and patient samples. Of note, [39] found an association between *GRIN2B* genotypes and CBCL/6-18 AP score in a general population sample aged 6-11. [8] investigated inattention and impulsive symptoms in a sample of ADHD children and found a positive correlation between both symptom classes and nine *GRIN2B* SNPs. On the same line, a study on attention performance in ADHD patients linked *GRIN2B* and GRIN2A variants to increased susceptibility to attention problems [12]. Our study confirms and strengthens these findings, showing a role of *GRIN2B* rs5796555-/A in the genetic risk for inattention/hyperactivity traits that remains stable during development.

### 4.2. *GRIN2B* Influence on Brain Structure

Brain imaging features may provide biological endophenotypes for genetic studies on inattention/hyperactivity. Nevertheless, in the growing imaging genetics field, just a few studies explored the link between *GRIN2B* markers and brain morphology. 

For the first time, we assessed the influence of six *GRIN2B* SNPs on a set of morphological features. The extraction of regional cortical thickness, volume and surface area has offered a unique opportunity to delineate the structural brain correlates of *GRIN2B* variants with high specificity.

Interestingly, we found selective associations between *GRIN2B* markers and morphological features. The brain feature that resulted in being most widely influenced by *GRIN2B* SNPs was regional GMV. On the contrary, the CT feature showed no influence of the *GRIN2B* markers. The minor allele of marker rs5796555-/A was associated with lower GMV in frontal, parietal and temporal regions. Conversely, the minor allele of marker rs2268119A/T was associated with lower CSA in left frontal and right occipital regions, and rs2216128G/C genotypes carrying the minor allele “C” showed CSA deficits in the right cingulate cortex.

Previous studies already showed an association between glutamatergic genes and regional GMV in children with neurodevelopmental disorders involving, at different levels, attention deficits. 

Probably due to different clinical populations and research methods, literature results are mixed. In patients with obsessive compulsive disorder (OCD), [40] reported *GRIN2B* SNPs to be associated with total thalamus volume. In another pediatric OCD study, a significant association between left orbitofrontal and right anterior cingulate volumes and *GRIN2B* SNPs emerged [23]. Of note, *GRIN2B* was linked to left posterior cingulate volume in adolescents with alcohol dependence, one of the disorders most closely related to impulsivity [24]. Since these findings emerged from clinical samples that share only some features with attention deficit syndromes, further investigations are needed to confirm our findings and interpret them in a wider dimensional perspective.

To our knowledge, the only imaging-*GRIN2B* study that has focused on inattention/hyperactivity is a resting state functional MRI (fMRI) study on ADHD children, which showed *GRIN2B* influence on regional homogeneity in left superior parietal cortex, being part of the attention circuit and with a role in inhibition [41].

Overall, these results suggest that *GRIN2B* regulation is not confined to specific brain regions but involves complex brain networks. Indeed, precuneus, cingulate, prefrontal, orbitofrontal, inferior parietal and temporal cortices, which were found to be affected by *GRIN2B* markers, are included in the default mode network (DMN) [42,43]. The DMN is a spontaneous resting state network that deactivates during task performance, whose activation has been implicated in attention and, specifically, in exteroceptive and interoceptive attentional orientation [44,45,46,47]. The DMN failure to deactivate during tasks might result in attentional intrusions and deficits in performance [48]. 

Moreover, the posterior and rostral cingulate cortex, prefrontal cortex, and inferior parietal lobule are part of the frontoparietal control network [49,50,51,52], involved in executive control. During tasks demanding direct attention to external information, activity increases in the frontoparietal control network and decreases in DMN [53]. 

The above evidence supports the hypothesis that *GRIN2B* effects on brain structure might be interpreted in terms of brain circuitries, especially those that in turn impact on behavioral functions. 

### 4.3. Brain Correlates of Attention/Hyperactivity Problems

In the imaging-behavioral analysis, we deliberately focused on the brain regions that resulted in being affected by *GRIN2B* rs5796555-/A, which may act as intermediate biological phenotypes in *GRIN2B* effect on inattention/hyperactivity. The study of the link between other brain features (e.g., regional CSA or CT) and attention/hyperactivity problems went beyond the scope of our study, but could be the subject of future investigations.

Interestingly, we found a negative association between CBCL/6-18 AP score and GMV in the left isthmus of the cingulum and the right inferior parietal cortex. The involvement of the posterior cingulate cortex and inferior parietal lobule in functional networks of attention control may explain the relation between attention problems and structural abnormalities in these areas and further supports the aforementioned network-based perspective.

### 4.4. From *GRIN2B* to Behavior through Neuroanatomy

In recent years, imaging genetics aimed to disentangle the pathways from genes to behavior. Instead of directly measuring the association between complex behavioral phenotypes and genetics, brain functionality and anatomy might be used as reliable intermediate phenotype, with a more direct and interpretable relation with genetics [41].

The results of our preliminary GLM analyses support the hypothesis that regional GMV (in left isthmus of cingulate cortex and right inferior parietal gyrus) might mediate *GRIN2B* effect on inattention/hyperactivity. Hence, two separate mediation analyses were performed to investigate the relationship among *GRIN2B* rs5796555-/A genotype, mean CBCL/6-18 AP score and, as mediator, GMV in left isthmus of cingulate cortex and right inferior parietal cortex. 

The failure to verify the second mediation hypothesis suggests that GMV in the right inferior parietal cortex, besides being regulated by *GRIN2B* rs5796555-/A, does not shape genetic susceptibility for inattention/hyperactivity. 

On the contrary, our results suggest that GMV in the left isthmus of cingulate cortex may play a key role in this mechanism. Indeed, after inclusion of this parameter in the mediation model, the direct effect of *GRIN2B* rs5796555-/A on CBCL/6-18 AP score became not significant, whereas its indirect effect through GMV in this region emerged to be significant. Specifically, more than 30% of the *GRIN2B* rs5796555-/A genotype effect on CBCL/6-18 AP score was mediated by GMV in left isthmus of cingulate volume. 

Therefore, we believe that this region might play a relevant role in translating *GRIN2B* variation to the complex attention phenotype. Until now, the isthmus of cingulate cortex, also known as retrosplenial cortex, has received little attention in studies on attention deficits. Nevertheless, a recent research found that the right cingulate isthmus was thinner in children with comorbid developmental coordination disorder (DCD) and ADHD compared to children with DCD alone. Previous fMRI studies reported an involvement of the retrosplenial cortex in spatial attention [54], episodic memory [55] and emotional processing [56]. Overall, this evidence enhances the need for focused research on structural and functional alterations of this region in attention/hyperactivity disorders.

### 4.5. Limitations

In the discussion of these results, the following limitations should be considered. First, the sample size was limited by difficulties related to the longitudinal study protocol. Specifically, the MRI acquisitions required high level of patient compliance, which was not always achieved due to the young age and clinical characteristics of our sample. The use of a modest sample has limited the statistical power and the reliability of the emerged imaging-genetic-behavioral associations, which need to be reproduced on larger, independent samples.

The integration of genetic, neuroimaging, and psychopathologic information enhanced the possible sources of error. Larger sample size replications are needed in order to minimize the risk of false positive results.

It is worth mentioning that our research protocol included children and adolescents with emotional and behavioral difficulties but not comparison subjects. Despite this limit, the clinical heterogeneity of the sample has enabled its stratification based on attention and hyperactivity problems and the investigation of the underlying genetic and neuroanatomic mechanisms.

Another important point is the lack of environmental factors in the model, which may have affected the findings’ reliability, as gene-environment interactions are implicated in the development of complex psychopathological behaviors [2,57]. 

Regarding the measurement of attention/hyperactivity problems, we used the CBCL/6-18 “Attention Problems” subscale, fulfilled by the participants’ caregivers. This scale evaluates both inattention and impulsivity from a hetero-referred point of view. In the future, it is desirable to integrate the measurement of these traits with neuropsychological tasks or clinical measures from different raters, and to disentangle the contributions of inattention and hyperactivity problems.

A final, intrinsic limitation of our study concerns the focus on a specific gene of the glutamatergic pathway. This choice was driven by growing evidence from genetic studies, suggesting a role of *GRIN2B* in attention deficits/hyperactivity traits. Given the increasing interest in polygenic risk factors, future study extensions should additionally consider the effect of other genes implicated in this behavior. We should investigate the interactions between genes, also known as “epistasis” [58], i.e., the extent to which the effect of one gene upon the phenotype is moderated by other genetic variations at a statistical and, possibly, a biological level [13].

## 5. Conclusions

Using a proof-of-concept imaging genetics mediation design, we explored the relationship between the glutamatergic *GRIN2B* gene variants and attention deficits and impulsive-hyperactive behaviors by introducing neuroanatomical measures as intermediate factors. 

Our findings confirm that brain anatomy, more specifically volumetry, is closely related to *GRIN2B* variations and can act as an intermediate phenotype between genetics and complex behaviors. The mediation results highlight a possible role of the left isthmus of the cingulum in mediating heritable risk for inattention/hyperactivity linked to *GRIN2B* variants. Confirmatory longitudinal studies are required to better delineate the genetic, neuronal and environmental mechanisms contributing to developmental risk pathways, with important implications for effective prevention, identification and treatment of early-onset psychiatric disorders.

## Figures and Tables

**Table 1:** Sociodemographic, clinical and cognitive characteristics of the sample.

	Males (n = 42)	Females (n = 16)	Total (n = 58)
**Wave 0**			
Age (years) ^1^	8.55 ± 2.51	9.38 ± 2.07	8.78 ± 2.43
SES ^1^	49.05 ± 18.59	36.25 ± 21.18	45.52 ± 20.17
CBCL Attention Problems ^1^	63.10 ± 9.40	66.56 ± 10.37	64.05 ± 9.80
FSIQ ^1^	108.81 ± 16.39	109.38 ± 13.93	108.96 ± 15.65
K-SADS-PL DIAGNOSIS ^2^			
ADHD	N = 9 (21.4%)	N = 4 (25.0%)	N = 13 (22.4%)
Any behavioral disorder	N = 10 (23.8%)	N = 0 (0.0%)	N = 10 (17.2%)
Any mood disorder	N = 12 (28.6%)	N = 6 (37.5%)	N = 18 (31.0%)
Any anxiety disorder	N = 19 (45.2%)	N = 9 (56.3%)	N = 28 (48.3%)
Other diagnoses	N = 7 (16.7%)	N = 5 (31.3%)	N = 12 (20.7%)
No current diagnosis	N = 2 (4.8%)	N = 1 (6.3%)	N = 3 (5.2%)
Comorbidities	1 diagnosis: N = 25 (59.5%)	1 diagnosis: N = 8 (50.0%)	1 diagnosis: N = 33 (56.9%)
	2 diagnoses: N = 13 (31.0%)	2 diagnoses: N = 5 (31.3%)	2 diagnoses: N = 18 (31.0%)
	3 diagnoses: N = 2 (4.8%)	3 diagnoses: N = 2 (12.5%)	3 diagnoses: N = 4 (6.9%)
**Wave 1**			
Age (years) ^1^	14.12 ± 2.09	15.58 ± 2.24	14.52 ± 2.23
CBCL Attention Problems^1^	59.48 ± 7.86	62.06 ± 7.24	60.19 ± 7.78
**Wave 2**			
Age (years) ^1^	15.80 ± 2.30	17.14 ± 2.43	16.17 ± 2.41
CBCL Attention Problems ^1^	57.83 ± 6.88	59.00 ± 4.98	58.16 ± 6.43

^1^ Mean ± standard deviation; ^2^ N (%). SES: socioeconomic status; FSIQ: full-scale intelligence quotient; K-SADS-PL: Kiddie schedule for affective disorders and schizophrenia for school-age children-present and lifetime version; ADHD: attention deficits/hyperactivity disorder. CBCL: Child Behavior Checklist.

**Table 2:** *GRIN2B* allele frequencies and Hardy-Weinberg equilibrium’s *p*-values.

*GRIN2B* SNP	Allele	Frequency ^1^	Hardy-Weinberg Equilibrium
rs5796555	-	0.71	0.201
	A	0.29	
rs1012586	G	0.66	0.744
	C	0.34	
rs2268119	A	0.73	0.213
	T	0.27	
rs2216128	A	0.74	0.146
	G	0.26	
rs11609779	C	0.84	0.546
	T	0.16	
rs2192973	G	0.78	0.115
	A	0.22	

SNP: single nucleotide polymorphism. ^1^ Fraction of the total. - is marker of the allele.

**Table 3:** General Linear Model results of *GRIN2B* markers effect on brain morphological parameters.

**GRIN2B** SNP	Allele	FS Feature	Brain Region	T ^1^	*p*	*p*_corr_ ^2^
rs5796555	“-/A” and “A/A”	Volume	Left inferior parietal	3.72	<0.001	<0.05
			Left isthmus cingulate	3.62	<0.001	<0.05
			Left middle temporal	3.90	<0.001	<0.05
			Left pars orbitalis	3.72	<0.001	<0.05
			Left precuneus	3.78	<0.001	<0.05
			Left rostral middle frontal	3.59	<0.001	<0.05
			Right caudal ACC	4.32	<0.0001	<0.01
			Right inferior parietal	4.14	<0.001	<0.01
			Right middle temporal	3.43	0.001	<0.05
			Right pars orbitalis	3.72	<0.001	<0.05
			Right rostral ACC	3.95	<0.001	<0.01
			Right rostral middle frontal	3.49	<0.001	<0.05
			Right transverse temporal	3.40	0.001	<0.05
rs2268119	“A/T” and “T/T”	Area	Left lateral orbitofrontal	3.40	0.001	<0.05
			Right lateral occipital	3.98	<0.001	<0.01
rs2216128	“G/C” and “C/C”		Right isthmus cingulate	3.42	0.001	<0.05

SNP: single nucleotide polymorphism; FS: FreeSurfer software; ACC: anterior cingulate cortex. ROI: region of interest. ^1^ general linear model design: ROI parameter ~ 1 + Gender + Age + *GRIN2B* SNP. Observations were 58, error degrees of freedom were 54. ROI volumes were normalized using total intracranial volume. ROI surface areas were normalized using total cortical surface area. ^2^ Level of significance after multiple comparison correction: *p* < 0.0016.

**Table 4:** Summary of mediation results.

Mediation Analysis 1				Mediation Analysis 2			
Parameter	Value	95% CI	*p*	Parameter	Value	95% CI	*p*
a	–0.45	[–0.26 0.26]	<0.001	a	0.48	[–0.26 0.26]	<0.001
b	–0.25	[–0.27 0.26]	0.037	b	−0.18	[–0.25 0.29]	0.08
c	0.31	[–0.27 0.26]	0.011	c	0.31	[–0.27 0.26]	0.011
ab	0.11	[–0.04 0.05]	<0.001	ab	n.e.	n.e.	n.e.
c’	0.20	[–0.27 0.27]	0.09	c’	n.e.	n.e.	n.e.

Causal variable X: **GRIN2B** marker rs5796555-/A. Outcome variable Y: CBCL/6-18 attention problem score. Mediator variable MA1: left isthmus of cingulate cortex volume. MA2: right inferior parietal cortex. CI: confidence interval. n.e: not evaluated.

## Data Availability

The data presented in this study are available on request from the corresponding author. The data are not publicly available due to privacy and ethical restrictions. Further information and requests for materials and methods should be directed to and will be fulfilled by the corresponding author.
